# Smear-Negative Pulmonary Tuberculosis with Cavitary Disease in a Young Immigrant Patient: A Diagnostic Challenge

**DOI:** 10.7759/cureus.90393

**Published:** 2025-08-18

**Authors:** Valentina Roa Forster, Macey Hedelund, Raul Munoz Franco, Jessica Espinosa, Jose Paz

**Affiliations:** 1 Internal Medicine, Florida International University, Herbert Wertheim College of Medicine, Miami, USA; 2 Internal Medicine, Nova Southeastern University Dr. Kiran C. Patel College of Osteopathic Medicine, Clearwater, USA; 3 Internal Medicine, Palmetto General Hospital, Hialeah, USA

**Keywords:** cavitary disease, diagnostic delay, immigrant health, latent tb reactivation, occupational risk, public health disparities, pulmonary infection, silica exposure, smear-negative, tuberculosis

## Abstract

Tuberculosis (TB) remains a public health concern in the United States, with most cases resulting from reactivation of latent infection. Smear-negative pulmonary TB (SNTB) presents a diagnostic challenge, often lacking classical radiographic findings or sputum smear positivity. Diagnosis in such cases depends on a high index of suspicion and the integration of clinical, radiographic, and epidemiologic information. We report the case of a 26-year-old male patient with no significant past medical history who presented with hemoptysis, fever, night sweats, and weight loss. He had immigrated from Cuba 18 months prior and worked in construction, with exposure to silica and aluminum dust. Chest imaging revealed cavitary pneumonia. Despite treatment for a presumed atypical pneumonia, his symptoms persisted. Immune-based testing ultimately supported a diagnosis of TB, and the patient improved after initiation of standard therapy. This case highlights the diagnostic complexity of SNTB, especially when radiographic findings are atypical and confounding serologic tests are present. His occupational exposure to silica likely contributed to disease susceptibility. He had experienced fever, cough, night sweats, weight loss, and hemoptysis for approximately one week prior to presentation, underscoring the importance of considering epidemiologic and occupational context in patients with acute respiratory symptoms. Early recognition of smear-negative disease is critical for timely treatment and public health control. As TB continues to affect urban U.S. areas with high immigration and occupational risk, early recognition of smear-negative disease is critical for timely treatment and public health control.

## Introduction

Tuberculosis (TB) can be difficult to diagnose, especially in smear-negative cases, which are often missed due to low bacillary load, atypical imaging, and misleading alternate diagnoses. [1]. These cases require a high index of suspicion and integration of epidemiologic, occupational, and clinical clues. Smear negativity typically reflects a lower bacillary burden, often seen in early or localized disease, and is associated with reduced sensitivity of sputum microscopy [2]. Furthermore, atypical radiographic findings and false-positive alternative diagnoses can further obscure recognition. TB pathogenesis involves inhalation of aerosolized *Mycobacterium tuberculosis*, which is phagocytosed by alveolar macrophages and contained within granulomas during the latent phase. Reactivation, often occurring years later, is driven by host or environmental factors that compromise immune containment [3]. These complexities underscore the importance of integrating clinical suspicion with epidemiologic, occupational, and radiologic contexts when evaluating patients with acute respiratory illnesses.

TB remains a significant public health concern in the United States, with the majority of cases being reactivation of latent TB infection rather than recent transmission [4]. The global incidence rate of TB is approximately 130 cases per 100,000 persons. Overall, the incidence of active TB in the United States has declined in recent decades; however, disparities persist, particularly among certain geographic regions. Florida is one of the four states (along with California, Texas, and New York) that collectively account for approximately half of all TB cases in the United States. It is one of the four states (along with California, Texas, and New York) that collectively account for approximately half of all TB cases in the United States. In 2023, Miami-Dade County reported a TB incidence rate of 4.9 per 100,000 persons compared to the national rate of 2.9 per 100,000, highlighting the concentration of cases in major urban areas and among populations born outside the United States. Miami-Dade County has historically reported rates exceeding this benchmark, underscoring the importance of increased clinical awareness and targeted public health efforts in these areas [5-8].

## Case presentation

We report the case of a 26-year-old male patient with no significant past medical history who presented to the hospital with hemoptysis. Prior to arrival at the hospital, the patient had one week of fever, chills, malaise, cough, night sweats, and five pounds of weight loss in the previous four days. The patient immigrated to the US one and a half years ago; however, he denied any recent travel. According to the patient, he had no prior history of TB, and he denied any known prior exposure to individuals with TB, recent incarceration, homelessness, or living in a crowded or shared house. The patient worked in construction and reports exposure to dust particles, including silica and aluminum, and denied ever smoking tobacco or using vaping products. Upon examination at the hospital, the patient was febrile, tachycardic, and had active hemoptysis.

**Figure 1:**
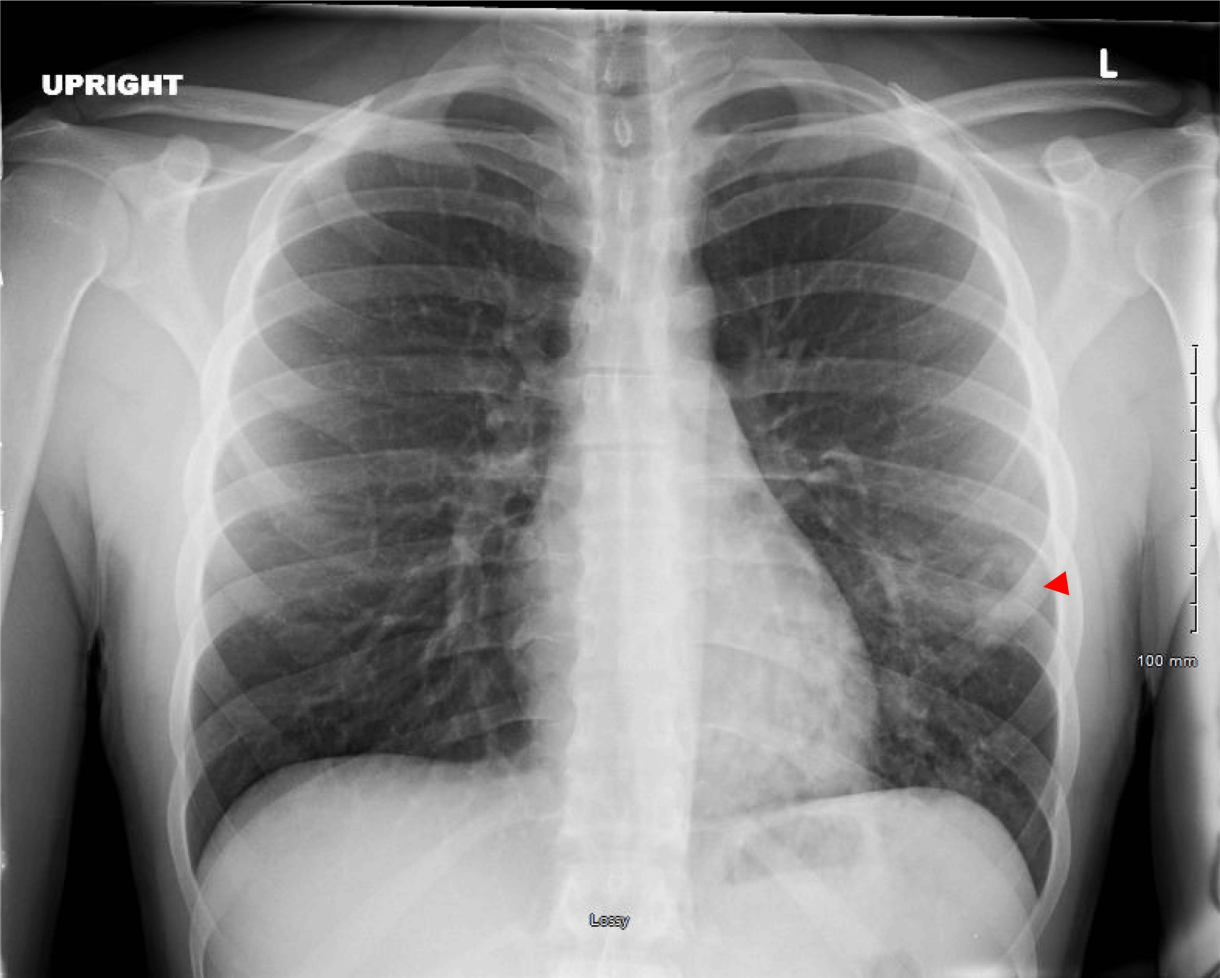
Portable One-View Chest X-ray (Anteroposterior View) Left lower lobe pneumonia with suspected cavitary lesion (red arrowhead) in the peripheral mid-to-lower left lung. Contrast-enhanced CT imaging of the chest was recommended for further evaluation.

**Figure 2:**
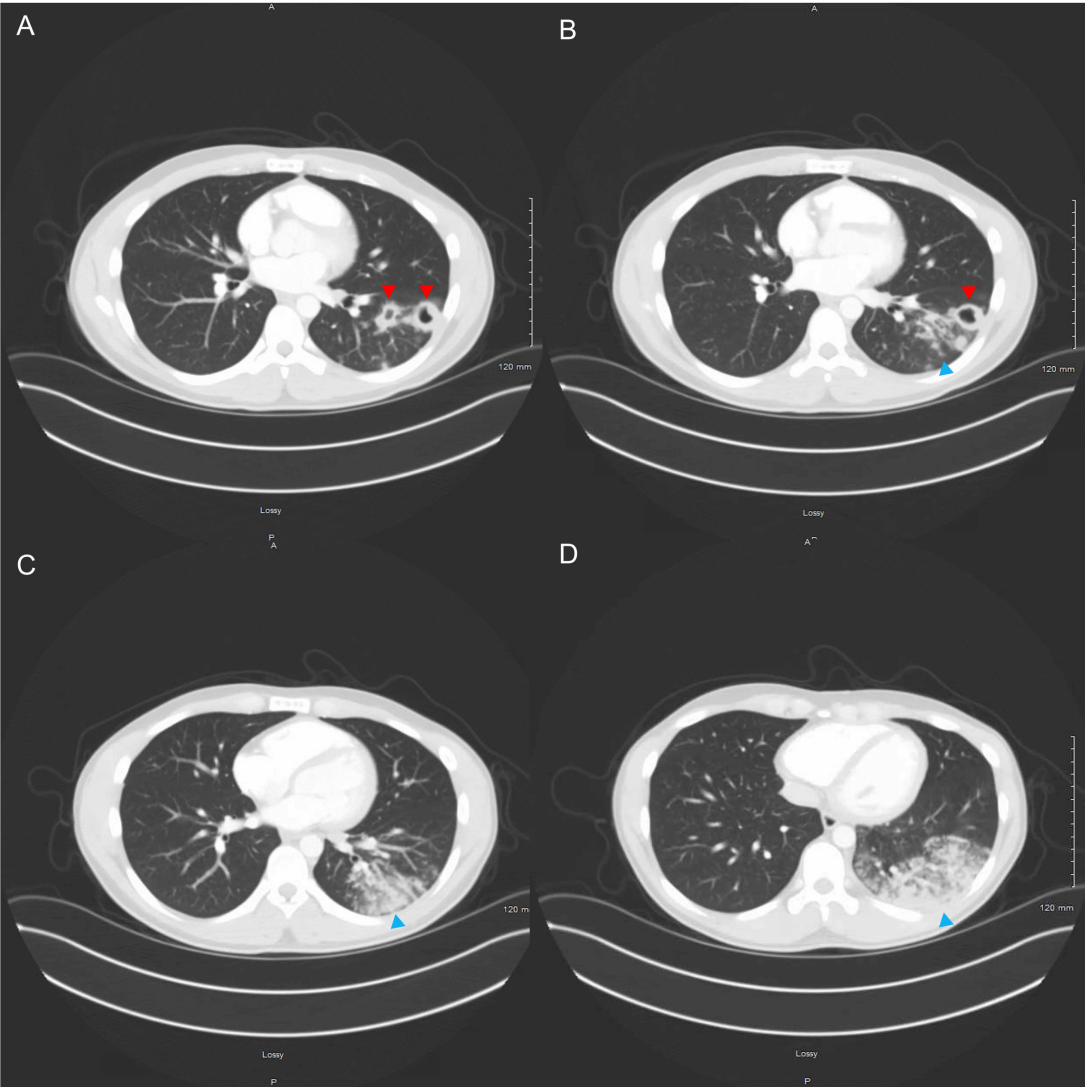
Axial CT Scan of the Chest With IV Contrast; Lung Window (Panels A–D, Cranial to Caudal) Cluster nodular and ground-glass density infiltrates with air bronchogram (blue arrowheads) and two small cavitations in the left lower lobe (red arrowheads). Thin-walled air-filled spaces in the left upper lobe, initially described as pneumatoceles, are visible. However, given the clinical context and eventual diagnosis of tuberculosis, these are favored to represent cavitations secondary to reactivation TB.

During admission, a chest X-ray showed left lower pneumonia with a suspected cavitary lesion in the peripheral mid-to-lower left lung (Figure 1). Computerized tomography (CT) of the chest revealed significant clusters of nodular and ground-glass density infiltrates with air bronchograms and two small cavitations in the left lower lobe, compatible with left lower lobe pneumonia and small pneumatoceles (Figure 2). Initial laboratory evaluation revealed a normal white blood cell count with relative neutrophilia. Hemoglobin and hematocrit levels were decreased, consistent with mild anemia. Red blood cell count was slightly below the normal range. Platelet count was normal with mildly decreased mean platelet volume. Red blood cell indices were overall unremarkable. The white blood cell differential demonstrated elevated neutrophil and monocyte percentages, with decreased lymphocyte levels and a low-normal absolute lymphocyte count (Table 1).

**Table 1:** Qualitative Summary of the Patient’s Hematologic, Microbiologic, Immunologic, and Infectious Disease Testing. Laboratory parameters are described in terms of increased, decreased, or normal relative to institutional reference ranges.

Test	Result	Units	Reference Range	Interpretation
Complete Blood Count (CBC)				
White Blood Cell Count	10	10³/μL	5.0 – 11.0	Normal
Hemoglobin	13.1	g/dL	14.0 – 18.0	Decreased
Hematocrit	43	%	42.0 – 52.0	Normal
Red Blood Cell (RBC)	4.27	10⁶/mm³	4.70 – 6.10	Decreased
Platelet Count	348	10³/μL	130 – 450	Normal
Mean Platelet Volume (MPV)	7.2	fL	7.4 – 10.4	Decreased
Mean Corpuscular Volume (MCV)	85	fL	80 – 94	Normal
Mean Corpuscular Hemoglobin (MCH)	29.1	pg	27.0 – 31.0	Normal
Mean Corpuscular Hemoglobin Concentration (MCHC)	34.6	g/dL	33.0 – 37.0	Normal
Red Cell Distribution Width (RDW)	13.3	%	11.5 – 14.5	Normal
White Blood Cell (WBC) Differential Count				
Neutrophils %	77.6	%	42.0 – 75.0	Increased
Lymphocytes %	13.4	%	20.0 – 50.0	Decreased
Monocytes %	10.2	%	0.0 – 9.0	Increased
Eosinophils %	1.5	%	0.0 – 7.0	Normal
Basophils %	0.4	%	0.0 – 2.0	Normal
Absolute Neutrophils	7.8	10³/μL	1.4 – 6.5	Increased
Absolute Lymphocytes	1.2	10³/μL	1.2 – 3.4	Low-Normal
Infectious Disease Workup				
QuantiFERON-TB Gold: Tuberculosis Antigen (TB Ag) – Nil 1	2.2	IU/mL	< 0.35	Increased
QuantiFERON-TB Gold: TB Ag – Nil 2	2.6	IU/mL	< 0.35	Increased
TB Test – Nil	0.34	IU/mL	–	Borderline
TB Test – Mitogen	>10.00	IU/mL	>0.5	Normal
Mycoplasma pneumoniae IgM	Positive	–	Negative	Increased
Influenza A & B Ag	Negative	–	Negative	Normal
SARS-CoV-2 Ag	Negative	–	Negative	Normal
Urine Streptococcus pneumoniae Ag	Negative	–	Negative	Normal
HIV 1/2 Antibody + P24 Antigen	Non-reactive	–	Non-reactive	Normal
Hepatitis B Surface Antigen	Not detected	–	Negative	Normal
Hepatitis C Antibody	Non-reactive	–	Non-reactive	Normal
Antinuclear Antibody (ANA), Cytoplasmic ANCA (cANCA), Perinuclear ANCA (pANCA)	Negative	–	Negative	Normal
Fungal Markers				
Beta-(1,3)-D-Glucan (Qualitative)	Not detected	–	Negative	Normal
Beta-(1,3)-D-Glucan (Quantitative)	<31.25	pg/mL	Negative <60	Normal

Autoimmune serologies, including antinuclear antibody (ANA), cytoplasmic ANCA (cANCA), and perinuclear ANCA (pANCA), were negative. The infectious disease workup was extensive and notable for a positive Mycoplasma pneumoniae IgM. The patient tested negative for hepatitis B surface antigen, hepatitis C antibody, HIV 1 and 2 antibody and p24 antigen, influenza A and B, SARS-CoV-2, and urinary *Streptococcus pneumoniae* antigen. Beta-(1,3)-D-glucan screening, including qualitative and quantitative assays, was negative (Table 1). Three consecutive sputum samples were negative for acid-fast bacilli (AFB). A computed tomography angiogram of the chest showed no active bleeding despite continued hemoptysis with clots. The patient was initially managed with broad-spectrum antibiotics, including vancomycin, piperacillin-tazobactam, and a completed course of azithromycin.

Further diagnostic testing included a positive QuantiFERON-TB Gold, indicating immune reactivity to *Mycobacterium tuberculosis*. While the Mantoux tuberculin skin test and Cy-TB were not performed, serial sputum samples were obtained for microbiologic evaluation. Initial sputum smears were negative for acid-fast bacilli; however, mycobacterial culture subsequently grew acid-fast bacilli prior to bronchoalveolar lavage (BAL). BAL specimens were negative on AFB smear but tested positive for *Mycobacterium tuberculosis *complex by polymerase chain reaction (PCR) test, and culture later confirmed the organism. Drug susceptibility testing was initiated in parallel, with no isoniazid resistance detected on KatG mutation analysis. The patient was empirically started on a standard four-drug regimen for drug-susceptible TB, including isoniazid, rifampin, pyrazinamide, and ethambutol, along with pyridoxine supplementation, given the high clinical suspicion, epidemiologic risk factors, and radiographic findings despite negative initial smears, and experienced progressive clinical improvement.

## Discussion

This case highlights the diagnostic challenge of smear-negative pulmonary TB (SNTB) in a young, otherwise healthy immigrant with significant occupational exposure. Despite presenting with classic symptoms of weight loss, fever, hemoptysis, and night sweats, three consecutive negative AFB smears complicated the diagnostic process. Regarding imaging, the initial chest CT described multiple thin-walled air-filled lesions as pneumatoceles, but the clinical context raised concern for pulmonary TB. Differentiating pneumatoceles from cavitations on imaging can be challenging, particularly early in the disease course. In this case, the radiographic appearance mimicked pneumatoceles, yet the evolution and associated findings supported a diagnosis of TB. The persistence of symptoms despite broad-spectrum antibiotic treatment, along with radiographic findings of left lower lobe cavitary pneumonia, raised concern for TB and prompted further testing, ultimately confirming the diagnosis.

The pulmonary consolidations seen in TB contain large amounts of necrotic material that harbor bacilli and drain through the airways. For these reasons, the number of AFB seen in a smear is closely correlated with the extent of consolidation [9]. SNTB comprises 10% to 61% of all TB cases. Cough, dyspnea, and hemoptysis are less frequently seen in SNTB than smear-positive TB (SMPT). Also, typical radiographic findings of upper lobe involvement from TB reactivation are not typically seen in SNTB [1]. These characteristics are thought to be a result of a smaller burden of mycobacteria in SNTB, which makes the diagnosis of TB challenging for physicians [2].

Our patient’s lower lobe cavitations may suggest primary active TB, which typically presents with lower lobe fibrocavitary disease. At the same time, reactivation TB typically presents with fibrocavitary disease involving the apical-posterior upper lobes or, less commonly, the superior segments of the lower lobes, due to the higher oxygen tension in those regions. However, radiographic patterns can overlap, especially in adults, and lower lobe involvement does not exclude reactivation, particularly in smear-negative disease [10]. The presence of cavitations is also associated with a higher mycobacterial burden and an increased risk of transmission [11]. Our patient’s three negative AFB smears despite the presence of two lower lobe cavitations is a finding that can be seen in a minority of patients with TB. Among the described mechanisms that can cause this discrepancy are poor sputum sample quality, low volume, or improper collection technique. Additionally, intermittent shedding of bacilli, localized disease with poor communication between the cavity and the airways, or partial airway obstruction can result in a low number of organisms in expectorated sputum despite radiographic cavitation [12,13].

Epidemiological risk factors must be taken into consideration along with the clinical picture. Recent immigration from countries with an elevated TB burden, such as our patient who immigrated from Cuba one and a half years prior to symptom onset, places patients at risk for reactivation of TB. The United States Preventive Services Task Force and the Centers for Disease Control and Prevention highlight that foreign-born individuals from higher-incidence countries account for the majority of TB cases in the U.S., with reactivation of latent TB being the predominant mechanism. The absence of other traditional risk factors such as incarceration, homelessness, or HIV does not diminish this epidemiologic risk, as birth or residence in a high-incidence country is itself a high-priority risk factor for both latent infection and reactivation [14, 15]. Clinicians should not exclude TB solely on the basis of negative smears and should pursue culture and nucleic acid amplification test (NAAT) and consider empiric treatment if suspicion remains high [12].

Occupational exposure to construction-related dust can contribute to a patient’s susceptibility to TB. Our patient had exposure to silica, which is known to impair alveolar macrophage function and disrupt pulmonary immune defenses, facilitating both the acquisition and reactivation of TB. Studies have confirmed that silica exposure without evidence of silicosis is independently associated with a significantly increased risk of active TB [16, 17]. On the other hand, our case was further complicated by the patient’s positive *Mycoplasma pneumoniae* IgM, raising concern for an alternative etiology. However, as false positives are common due to cross-reactivity and nonspecific IgM elevation, these findings must be interpreted with caution. The American Thoracic Society (ATS), Centers for Disease Control and Prevention (CDC), and Infectious Diseases Society of America (IDSA) advise clinicians to avoid diagnostic anchoring on serologic data of limited specificity and maintain a high suspicion for TB in patients with compatible symptoms and risk factors, even when AFB smears are negative [18].

The setting of this case is Miami-Dade County, and it reflects a broader national concern. Miami-Dade County consistently reports higher TB incidence than both Florida and the United States overall, a trend largely driven by its sizable foreign-born population, which comprises over half of residents and includes many from TB-endemic countries [19,20]. However, Miami-Dade is not unique. Other metropolitan areas with similar immigration patterns, socioeconomic disparities, and occupational exposures are likely to experience comparable epidemiologic pressures. As population mobility increases and latent TB reservoirs persist in under-screened groups, clinicians across the U.S., particularly in urban and coastal cities, must maintain vigilance for TB reactivation. This case emphasizes that, even in low-incidence countries, the risk of delayed diagnosis remains high if the epidemiologic context is not carefully considered [21].

## Conclusions

This case demonstrates how pulmonary TB can evade early detection when classic diagnostic tools such as AFB smears and initial imaging are inconclusive. Despite the presence of hallmark TB symptoms, the diagnosis was initially obscured by negative smears, lower lobe cavitation, and a confounding positive *Mycoplasma pneumoniae* IgM. The patient’s recent immigration from a TB-endemic region, combined with occupational exposure to silica, formed a high-risk backdrop that might have been overlooked without careful attention to epidemiologic and occupational factors. Clinicians must remain vigilant for smear-negative TB, particularly in patients from high-incidence regions, those with relevant workplace exposures, or those presenting with persistent respiratory symptoms despite empiric antibiotic therapy. Early consideration of TB in such patients can expedite treatment, prevent complications, and reduce community transmission. As TB reemerges in high-risk urban settings, this case reinforces the need for an integrated clinical and public health approach that considers not only symptoms but also the social and environmental context in which the disease occurs.
